# Informed consent in field trials of gene-drive mosquitoes

**DOI:** 10.12688/gatesopenres.12771.1

**Published:** 2017-12-11

**Authors:** Pamela A. Kolopack, James V. Lavery

**Affiliations:** 1 Dalla Lana School of Public Health, and Joint Centre for Bioethics, University of Toronto, Toronto, ON, M5T 3M7, Canada; 2 Hubert Department of Global Health, Rollins School of Public Health, and Center for Ethics, Emory University, Atlanta, GA, 30322, USA

**Keywords:** informed consent, research ethics, gene-drive, genetically-modified mosquitoes, field trials, global health

## Abstract

The US National Academies’ (NAS) recent report ‘Gene Drives on the Horizon: Advancing Science, Navigating Uncertainty, and Aligning Research with Public Values’ examines the requirements of responsible conduct in research involving gene drives in non-human organisms. Many of the complex ethical issues raised by the introduction of gene drive technologies for mosquito population control have been anticipated during the development and field-testing of earlier-generation genetic engineering approaches with mosquitoes. One issue—the requirement for informed consent in field trials—is not addressed explicitly in the NAS’ report. Some commentators have presumed that informed consent should play a role as a protection for research participants in studies of genetically modified mosquitoes. Others have argued that there are no human subjects of field trials, so the informed consent requirement does not apply. It is both ethically and practically important that these presumptions are adequately scrutinized to ensure that any applications of informed consent in these trials are properly justified. We argue that informed consent from individual research participants in gene drive trials may be required: (1) when blood and other forms of clinical data are collected from them, as will likely be the case in some studies involving epidemiological endpoints, such as the incidence of new infections with dengue and malaria; (2) when they participate in social science and/or behavioral research involving the completion of surveys and questionnaires; or (3) when their home or property is accessed and the location recorded as a spatial variable for the release or collection of mosquitoes because the precise location of the household is important for entomological reasons and these data constitute identifiable private information at the household level. Importantly, most regulations and guidelines allow these requirements to be waived or modified, to various degrees, according to the judgment of Institutional Review Boards.

## Introduction

The US National Academies’ (NAS) recent report ‘Gene Drives on the Horizon: Advancing Science, Navigating Uncertainty, and Aligning Research with Public Values’ ^1^ examines the requirements of responsible conduct in research involving gene drives in non-human organisms. One of the most promising applications of gene drive technologies on the horizon is the modification of disease-transmitting mosquitoes in attempts to diminish their populations or compromise their capacities as vectors. Many of the complex ethical, social, and cultural issues raised by the introduction of gene drive technologies for mosquito population control have been anticipated during the development and field-testing of earlier-generation genetic engineering approaches with mosquitoes ^2– 5^. These studies are complex and require unique designs ^6^ involving various combinations of outcome measures and a progression of activities—from purely entomological studies to characterize effects on mosquito populations ^7^ to studies involving the measurement of epidemiological outcomes associated with modified mosquitoes in defined areas.

Extensive media coverage of these early approaches has thrust the many challenges associated with testing these new technologies squarely into the public spotlight. One issue—the requirement for informed consent in field trials—is not addressed explicitly in the NAS’ report ^1^, but has gained international attention most recently around a planned field trial of genetically modified *Aedes aegypti* mosquitoes to prevent Zika virus transmission in Florida. The proposed field trial sparked considerable controversy. In the run-up to a Monroe County, Florida, ballot measure about the proposed trial in the US general election on November 8, 2016 ‘No Consent’ signs dotted the landscape in Key Haven, Florida ^8^, the proposed site of the planned trial. In addition, almost 170,000 people signed a petition that, among other things, claimed that the planned releases will be conducted “against the wishes of the locals and the scientific community” ^9^. Conflicting votes on the ballot measure across Monroe County on November 8 stalled a decision by the Florida Keys Mosquito Control District Board of Commissioners about the fate of the proposed trial ^10^, and a subsequent meeting of the Board saw community members claiming human rights violations if the proposed release trial were to be conducted without the informed consent of residents ^11^.

## Conflicting presumptions about the role of informed consent

Even though the genetically modified mosquitoes in the proposed Florida trial did not involve gene drives, the response to their planned introduction in open field trials foreshadows some of the issues that are likely to be even more vigorously contested in future trials involving gene-drive mosquitoes and other insects. Some commentators have presumed that informed consent should play a role as a protection for research participants in studies of genetically modified mosquitoes ^4^, under-pinned by competent regulatory oversight and robust community engagement ^5^. This presumption has been reinforced in some early experiences with the testing of biologically-modified mosquitoes, in which regulators insisted on household level informed consent as a condition of approval for open-release trials ^12^. Other commentators have argued that “(t)here are, strictly speaking, no human subjects of field trials, so the regulations governing human subjects research, which require informed consent from every participant, do not apply.” ( 13, p. 716) It is both ethically and practically important that these presumptions are properly scrutinized to ensure that any appeals to apply informed consent in future trials of genetically modified mosquitoes constitute an appropriate response to the ethical stakes involved, and not simply a reflexive appeal to the most familiar tool in the research ethics toolbox.

Informed consent to participate in research is fundamentally a way for individuals to authorize researchers to perform various research-related actions that would otherwise constitute some form of violation of the individual’s rights. For example, administering an experimental drug to someone in a research study without their consent would normally constitute battery. But because the central actions of researchers in research involving genetically-modified and gene-drive mosquitoes—the release, tracking, and collection of mosquitoes—are not targeted at individuals, a key question for all of these trials is who should be considered a research subject, and under what conditions informed consent should be applied as a protection.

### Who is a human research subject?

These questions have caused confusion in several complex research designs—in particular, cluster-randomized trials—and in response McRae *et al.* ^14^ have proposed a general definition of a human research subject based on their analysis of the common elements of informed consent represented in international research ethics guidelines and influential policies, including the US Common Rule regulations governing research with human subjects. Their analysis remains consistent with the recent ‘Final Rule’ revisions of the Common Rule ( https://www.hhs.gov/ohrp/regulations-and-policy/regulations/finalized-revisions-common-rule/index.html). According to their definition, “a human research subject is an individual whose interests may be compromised as a result of interventions in a research study” ^14^. By “interventions”, McRae *et al.* ^14^ refer both to the experimental procedure being investigated as well as to non-experimental data collection procedures. More specifically, a human research subject is an individual: 1) who is directly intervened upon by an investigator as either (a) a recipient of a study intervention or (b) as someone who undergoes non-experimental interventions to collect data; 2) who is deliberately intervened upon via manipulation of the individual’s environment by the investigator in such a way as to have a direct effect on the individual; 3) who communicates or has interpersonal contact with an investigator for the purpose of collecting data through, for example, interviews, focus groups, or questionnaires; and 4) about whom an investigator obtains identifiable private information for the purpose of collecting data ^14^.

Given the nature of the interventions, it does not seem plausible to claim that any individual at or near a release site will be “directly intervened upon” by an investigator as a recipient of a study intervention (criterion 1(a) above) or “deliberately intervened upon via manipulation of the individual’s environment” ^14^ (criterion 2 above). Most release trials will involve male mosquitoes, which do not bite humans, and the mosquitoes will bring about the hoped-for population suppression or replacement through competition for reproductive opportunities with wild-type mosquitoes, rather than through deliberate interactions with humans.

### Appropriate applications of informed consent

In line with the definition of McRae *et al.* ^14^, we believe individuals satisfy the conventional requirements to be considered human subjects in research with genetically modified mosquitoes in the following circumstances: (1) when blood and other forms of clinical data are collected from them, as will likely be the case in some studies involving epidemiological endpoints, such as the incidence of new infections with dengue and malaria; (2) when they participate in social science and/or behavioral research involving the completion of surveys and questionnaires; or (3) when their home or property is accessed and the location recorded as a spatial variable for the release or collection of mosquitoes because the precise location of the household is important for entomological reasons and these data constitute identifiable private information at the household level.

Importantly, it also does not follow that the normal requirements may not be waived or modified according to the judgment of research ethics committees or institutional review boards, as most regulations and guidelines anticipate, and allow, to various degrees. Our circumstance (3) above, may be better understood as more general requests for permission and gestures of common respect and decency, governed by social convention and relevant laws related to privacy and trespass, rather than applications of informed consent to protect research subjects.

Living in the vicinity of a release trial does not automatically render someone a research subject and therefore it is inappropriate to require informed consent from every individual in the vicinity simply because the technologies being deployed are still in their testing and development stages. Arbitrarily requiring informed consent from every individual and household in geographic proximity to a release trial misrepresents and undermines the value of informed consent in research and establishes worrisome precedents about the appropriate application of research ethics policies and procedures. It also raises potentially insurmountable logistical challenges that will ultimately impede important science, with no clear ethical rationale.

Field trials of gene drive and other genetically modified mosquitoes do not fit neatly into our current regulatory definitions of clinical trials—including the National Institutes of Health’s recent revision to its definition of clinical trials, a definition that distinguishes between clinical trials and other types of clinical research and automatically triggers a set of regulatory procedures, including individual informed consent from human subjects ( https://grants.nih.gov/grants/guide/notice-files/NOT-OD-15-015.html). And concern about an overly expansive definition of “clinical trials” also figured prominently in the public comments on the recently adopted ‘Final Rule’ revisions ( https://www.gpo.gov/fdsys/pkg/FR-2017-01-19/pdf/2017-01058.pdf). As a result, there is likely to be considerable debate about the appropriate regulatory standards for these trials, particularly as the trials begin to incorporate epidemiological endpoints. It is also likely that funders and regulatory agencies will seek refuge in familiar policy tools, whether or not they represent the most appropriate response to the specific challenges at hand.

## Conclusion

What constitutes fair and legitimate authorization for field trials of gene drive and genetically modified mosquitoes is a critically important question ^15^, and ensuring individual informed consent to specific research processes and procedures surely has a role to play in the overall balance. But it is a narrow role and should not deflect attention from the more complex governance challenges of developing the appropriate regulatory regimes and authentic stakeholder engagement.
