# Supplementary material for: Informed consent in field trials of gene-drive mosquitoes
Source: Gates Open Res. 2017 Dec 11;1:14. [Version 1] doi: 10.12688/gatesopenres.12771.1 (PMC5757819; doi:10.12688/gatesopenres.12771.1)
Supplement: Peer review history

# Peer review: Referee response for version 1

**DOI:** 10.21956/gatesopenres.13831.r26149

**Published:** 2018-01-08

**Authors:** Cinnamon S. Bloss, Cynthia E. Schairer

**Affiliations:** 1 Department of Medicine, University of California San Diego, La Jolla, CA, USA; 2 Department of Family Medicine and Public Health, UC San Diego School of Medicine, La Jolla, CA, USA

Kolopack and Lavery argue that informed consent in field trials of gene-drive mosquitoes should be applied only when individuals satisfy the conventional requirements to be considered human subjects in research, i.e., when there is effectively a reasonable expectation of individual risk. Defining the circumstances under which field trials require informed consent is useful; it carves out a practical space for informed consent to be used in field trials of different designs and, as the authors point out, avoids “misrepresent[ing] and undermin[ing] the value of informed consent.” In the final sentence of the piece, the authors acknowledge the limitations of informed consent as a tool for coping with community-level risks by calling for serious attention to regulatory regimes and stakeholder engagement, but this message is obscured by three aspects of the article that want clarification.

 First, the abstract concludes with a somewhat different message than the body of the piece, pointing to the allowance of informed consent requirements to be waived or modified by Institutional Review Boards (IRB). The wording is ambiguous, but might be read as a complaint or warning that stronger informed consent requirements are not in place. Or perhaps it is meant to be a reminder that informed consent is not always sacrosanct, as the requirement may be waived by an IRB. But the argument made in the body of the article focuses on a cogent proposal for the proper limitations of informed consent in the context of field trials and ends with a late reminder of other, critically important “governance challenges”, that need to be met for “fair and legitimate” field trials to go forward.

 Second, the final criteria for when consent may be required – when property is accessed and location recorded – requires further consideration, in our estimation. The authors argue that this information is both private and identifiable and that if collected, would therefore meet criteria for human subjects research and require individual informed consent. We question, however, the extent to which there is a reasonable expectation of privacy when a property is accessed in the manner the authors describe.  Specifically, property is accessed and recorded in this way when homes are bought and sold and in the name of other public utilities, goods, and services (e.g., provision of clean water, and even environmental mosquito control services). Therefore, merely recording entomological data based on observations made at a specific location or on a person’s property, may not constitute a scenario in which there is a reasonable expectation of privacy. In fact, the authors also seem to acknowledge this in a later paragraph in their article when they state that this “may be better understood as more general…gestures of common respect and decency,” but further discussion of this point is warranted.

 Finally, as Kolopack and Lavery suggest at the end of their article, informed consent guidelines should not overshadow the challenges of developing “appropriate regulatory regimes and authentic stakeholder engagement.” It is worth mentioning that in the case of the Florida Oxitec trial, a regulatory mechanism was in place – though arguably insufficient – but the process did little to quell anxiety about the unknown consequences of the intervention to be tested among the vocal minority of residents in Key Haven who were opposed to the trial. The appeal to informed consent, then, could be construed as an attempt to wield individual power over a collective mechanism (and an apparently successful attempt as the proposed Oxitec trial has been postponed until a different trial site is identified). Though the authors do not claim this, we believe it warrants mention that guidelines about when to require informed consent cannot address the underlying issue at stake in the protests against the Florida Oxitec trial. Ultimately, local protestors did not trust Oxitec, the Food and Drug Administration, or the local mosquito control board to adequately manage community-level risk associated with the trial. Thus, despite the provocative language of informed consent, Florida residents and activists were concerned about unintended consequences secondary to a release – risks that cannot be managed with informed consent.

 The authors also have an opportunity to mention the existing structures in place to help us make collective choices about how to act in the face of such risks. Specifically, regulatory bodies are set up to have experts assess risks before approving field trials so that the lay public will not be asked to evaluate risk without the necessary expertise or accept unreasonable risks (see for example, epa.gov). For instance, like the risks of pesticides, the risks associated with field trials of genetically engineered insects cannot be managed by individuals in the ways that risks associated with blood draws or surveys may be. Nor is it appropriate to react to the protests of some with outright rejection of a technology with great potential to protect public health. There certainly needs to be greater clarity on and agreement in the field regarding the “rules” of informed consent for field trials, and we applaud Kolopack and Lavery for their proposal in this regard. We also note, however, that a focus on informed consent in the landscape of field trials of gene-drive mosquitoes and other insects runs the risk of allowing a vocal minority to set the terms of the debate, obscuring the need for transparent and independent regulatory structures and effective two-way communication between researchers and the communities they wish to help.

We have read this submission. We believe that we have an appropriate level of expertise to confirm that it is of an acceptable scientific standard.

# Peer review: Referee response for version 1

**DOI:** 10.21956/gatesopenres.13831.r26148

**Published:** 2018-01-03

**Authors:** George J. Annas

**Affiliations:** 1 Center for Health Law, Ethics & Human Rights (CLER), Boston University School of Public Health, Boston, MA, USA

Time to declare informed consent to field trails of gene-drive mosquitoes a dead issue.

 George J. Annas

 Kolopack and Lavery (1) are correct both to suggest that the NAS report on “Gene Drives on the Horizon” (2) is the most authoritative report on the subject to date, and that the report did not “explicitly” address informed consent of the people who live in the area in which genetically-modified mosquitoes are released for study. Nonetheless, I think it is time to call an end to what’s left of the informed consent debate in this context. This is not only, as others have written, because “informed consent is neither ethically required nor practically feasible,”(3) but most centrally because what is at issue *is* ethically required and practically necessary: community engagement (sometimes referred to as community consultation). The NAS gene drives report, for example, quotes an earlier (1996) National Research Council report as saying, “The normative rationale [for broad participation in risk decisions] derives from the principle that government should obtain the consent of the governed. Related to this principle is the idea that citizens have rights to participate meaningfully in public decision making and to be informed about the bases for governmental decisions.” (p. 133)  Immediatly after the quotation, the gene drives report goes on: “…engagement enhances transparency and ensures some level of meaningful participation and consent.” It is fair to say that just what “meaningful participation” means can vary from community to community, as well as from one “public” to another, and is the subject of on-going debates. It is also fair to say, I think, that the notion that “consent” means “informed consent” from individuals at potential risk from mosquito release experiments is not suggested in the gene drives report, and, I think, it is time to declare informed consent in this context dead. 

 Kolopack and Lavery may seem to disagree with this unequivocal conclusion, but I’m not convinced that they do. (The Vietnam example of regulators insisting on “household level informed consent as a condition of approval for open-release trials” is not a counter example, as there is no such thing as “household level informed consent,” just individual informed consent.[4])  Kolopack and Lavery suggest only 3 instances in which members of the population living in an area where modified mosquitoes will be released could qualify as “research subjects” and hence as people whose informed consent may have to be sought: (a) when blood or similar biologic samples are collected from individuals for the study; (2) when individuals are asked to respond to questionnaires or surveys; and (3) when the home or property of individuals is being assessed to obtain data relevant to the research. There are two points to be made about this list. The first is made by Kolopack and Lavery themselves, who observe that even if one accepts this list in its entirety, “it does not follow that the normal requirements may not be waived or modified according to the judgment of…institutional review boards [IRBs]…”  Anticipating waiver of consent must mean, it seems to me, that the authors do not think formal informed consent is ethically-required in these circumstances. More centrally, however, is that the first two examples, blood draws and survey responses, are interventions routinely dealt with by IRBs, and the context of mosquito-release studies present no novel or unique ethical issues.(5) Finally, number 3, entry into one’s home or property, is something routinely done by friends, delivery persons, and even public officials, and requires only the oral agreement or authorization of the inhabitant, as Kolopack and Lavwery themselves point out.

 Work on this letter was partially funded by DARPA, Contract No. HR0011-17-2-0042, Controlling and Countering Gene Drives in Mosquitoes.

I have read this submission. I believe that I have an appropriate level of expertise to confirm that it is of an acceptable scientific standard.

## References

[1] KolopackPALaveryJV: Informed consent in field trials of gene-drive mosquitoes [version 1; referees: 2 approved]. *Gates Open Res*.2017;1(14) : 10.12688/gatesopenres.12771.1 10.12688/gatesopenres.12771.1 29355214PMC5757819

[2] National Academies of Sciences, Engineering, and Medicine: Gene drives on the horizon: advancing science, navigating uncertainty, and aligning research with public values. *National Academies Press*.2016; 10.17226/23405 10.17226/23405 27536751

[3] NeuhausCPCaplanAL: Ethical lessons from a tale of two genetically modified insects. *Nat Biotechnol*.2017;35(8) : 10.1038/nbt.3927 713-716 10.1038/nbt.3927 28787406

[4] McNaughtonDDuongTT: Designing a community engagement framework for a new dengue control method: a case study from central Vietnam. *PLoS Negl Trop Dis*.2014;8(5) : 10.1371/journal.pntd.0002794 e2794 10.1371/journal.pntd.0002794 24853391PMC4031131

[5] AnnasGJ: Beyond Nazi War Crimes Experiments: The Voluntary Consent Requirement of the Nuremberg Code at 70. *Am J Public Health*.2018;108(1) : 10.2105/AJPH.2017.304103 42-46 10.2105/AJPH.2017.304103 29161060PMC5719679

# Peer review: Referee response for version 1

**DOI:** 10.21956/gatesopenres.13831.r26146

**Published:** 2017-12-21

**Authors:** Carolyn P. Neuhaus

**Affiliations:** 1 The Hastings Center, Garrison, NY, USA

Kolopack & Lavery outline conditions under which persons involved in research on gene-drive mosquitoes may also be considered “human subjects of research.” They identify three conditions under which informed consent from individual research participants in gene-drive trials may be required: (1) when blood and other forms of clinical data are collected from them; (2) when they participate in social science and/or behavioral research involving the completion of surveys and questionnaires; or (3) when their home or property is accessed and the location recorded as a spatial variable for the release or collection of mosquitoes.

 I don’t think anyone would take issue with (1) or (2). I might suggest distinguishing, though, between research activities undertaken as part of a field trial of gene-drive mosquitoes, which might study primarily ecological effects, and activities undertaken as part of subsidiary epidemiological or behavioral research on the impact of field trials on human health or well-being. The former may proceed without the latter. Field trials with epidemiological endpoints may collapse the distinction between ecological research and epidemiological research; though study design and choice of endpoints in field trials also merit closer ethical analysis.

 Kolopack & Lavery’s condition (3) will require a bit more development I think. The stated rationale for (3) is, “the precise location of the household is important for entomological reasons and these data constitute identifiable private information at the household level.”

 While I can appreciate that it would be wise, if not also necessary, to ask for permission to access someone’s property for the purpose of data collection, it is debatable whether that act renders the occupant a “human subject of research.” It seems somewhat strange that knowledge of the location of a release/catch site or its specific address constitutes “identifiable private information.” I know that there is a house at 10 Main Street, but that tells me nothing about its occupants. To find out that mosquitoes were released at 10 Main Street provides no further information about them -- except, perhaps, that the occupants of that address agreed to allow the release of gene-drive mosquitoes on their property. Does this constitute “identifiable private information”? Perhaps additional private information would be collected; it’d be worth specifying what in order to clarify which data constitute identifiable private information such that its collection would require informed consent.

 The authors further state that it does not follow from their assertion that home dwellers may be human subjects of research that informed consent, in the legal sense, would be required of them prior to the release of insects on their property. They say that instead, “general requests for permission and gestures of common respect and decency” could substitute obtaining informed consent to research participation. But the rationale for this substitution is lacking. Perhaps this gets to the point that one should ask permission before entering someone’s property, but this is not the same thing as obtaining informed consent to research participation.

 Research ethics committees considering whether to waive or modify informed consent requirements for field trial research will likely need further guidance if they find themselves debating these matters. Kolopack & Lavery ask the right questions and make an excellent contribution to discussion about the rationale for informed consent in field trials of insects and animals with gene drives. I look forward to continued conversation.

I have read this submission. I believe that I have an appropriate level of expertise to confirm that it is of an acceptable scientific standard.

# Peer review: Referee response for version 1

**DOI:** 10.21956/gatesopenres.13831.r26147

**Published:** 2017-12-21

**Authors:** Jason A. Delborne

**Affiliations:** 1 North Carolina State University, Raleigh, NC, USA

This open letter considers the manner in which informed consent might apply to trials of genetically modified (GM) or gene drive modified (GDM) mosquitoes during field trials. At issue are two important questions: 1) when should we consider humans who live near field trial sites as "human subjects," thus triggering requirements for informed consent, and 2) how well does "informed consent" capture ethical and normative requirements for the authorization of GM or GDM mosquito field trials?

 The three criteria posed for when "individuals satisfy the conventional requirements to be considered human subjects in research with genetically modified mosquitoes" (p. 4) are reasonable and defensible, and they answer question #1 above. The first criteria - "when blood and other forms of clinical data are collected from them" (p. 4) - could be highly relevant during field trials of mosquitoes whose sponsors seek to make an epidemiological claim about the organism. In the U.S. regulatory context, such data will be required if the Food and Drug Administration is measuring, for example, whether dengue incidence decreases with the release of the mosquitoes. Claims solely about population suppression may not require such data. The second criteria - "when they participate in social science and/or behavioral research" (p. 4) - will likely be triggered with such high levels of interest by social scientists in GM and GDM field trials. Of note, however, such social science research may not always be well integrated into field trial research, suggesting only that the collection of typical social science data will require typical human subjects informed consent. The third criteria - related to the collection of precise household location data - is a thoughtful extension of criterion #2, as issues of identity and privacy are well-worn modes of concern in social science research.

 The authors also provide some perspective on the question of whether "informed consent" is the right lens for discerning ethical and responsible research in GM and GDM field trials. Importantly, they gesture to their paper on community engagement (ref #15) and also state that "ensuring individual informed consent...is a narrow role and should not deflect attention from the more complex governance challenges of developing the appropriate regulatory regimes and authentic stakeholder engagement" (p. 4). In other words, while understanding how informed consent applies to GM and GDM field trials is important, it is not sufficient. From my perspective as a reviewer, this claim is at least as important as the identification of criteria 1-3 (described above) and somewhat unfortunately buried in the conclusion. The authors discussion of the case study of the Florida Keys Mosquito Control District would be an excellent place to explicitly acknowledge how questions of informed consent are very unlikely to capture the complex normative and political dimensions that will accompany at least the early years of GM and GDM organism field trials. As the authors note, public debates often map onto existing regulatory structures - hence the claims in Florida of human rights violations associated with a lack of informed consent. Such mapping reflects issues of power and authority in specific contexts - for example, the ability of community members or stakeholders to have a voice in the authorization of a field trial. Clarifying the specific application criteria of informed consent - the focus of this paper - may do little to improve the quality of debate unless and until the development of "appropriate regulatory regimes and authentic stakeholder engagement" (p. 4), which remains a rich area for research and public discourse.

I have read this submission. I believe that I have an appropriate level of expertise to confirm that it is of an acceptable scientific standard.
